# Silicon–van der Waals heterointegration for CMOS-compatible logic-in-memory design

**DOI:** 10.1126/sciadv.adk1597

**Published:** 2023-12-08

**Authors:** Mu-Pai Lee, Caifang Gao, Meng-Yu Tsai, Che-Yi Lin, Feng-Shou Yang, Hsin-Ya Sung, Chi Zhang, Wenwu Li, Jun Li, Jianhua Zhang, Kenji Watanabe, Takashi Taniguchi, Keiji Ueno, Kazuhito Tsukagoshi, Ching-Hwa Ho, Junhao Chu, Po-Wen Chiu, Mengjiao Li, Wen-Wei Wu, Yen-Fu Lin

**Affiliations:** ^1^ Department of Materials Science and Engineering, National Yang Ming Chiao Tung University, Hsinchu 30010, Taiwan.; ^2^ Shanghai Frontiers Science Research Base of Intelligent Optoelectronics and Perception, Institute of Optoelectronics, Fudan University, Shanghai 200433, China.; ^3^ Department of Physics, National Chung Hsing University, Taichung 40227, Taiwan.; ^4^ Institute of Electronics Engineering, National Tsing Hua University, Hsinchu 30013, Taiwan.; ^5^ State Key Laboratory of Photovoltaic Science and Technology, Department of Materials Science, Fudan University, Shanghai 200433, China.; ^6^ School of Microelectronics, Shanghai University, Jiading, Shanghai 201800, China.; ^7^ Research Center for Electronic and Optical Materials, National Institute for Materials Science, 1-1 Namiki, Tsukuba 305-0044, Japan.; ^8^ Research Center for Materials Nanoarchitectonics, National Institute for Materials Science, 1-1 Namiki, Tsukuba 305-0044, Japan.; ^9^ Department of Chemistry, Graduate School of Science and Engineering, Saitama University, Saitama 338-8570, Japan.; ^10^ International Center for Materials Nanoarchitectonics, National Institute for Materials Science, Tsukuba 305-0044, Japan.; ^11^ Graduate Institute of Applied Science and Technology, National Taiwan University of Science and Technology, Taipei 106, Taiwan.; ^12^ Department of Material Science and Engineering, Institutes of Nanoscience, i-Center for Advanced Science and Technology (i-CAST), National Chung Hsing University, Taichung 40227, Taiwan.

## Abstract

Silicon CMOS-based computing-in-memory encounters design and power challenges, especially in logic-in-memory scenarios requiring nonvolatility and reconfigurability. Here, we report a universal design for nonvolatile reconfigurable devices featuring a 2D/3D heterointegrated configuration. By leveraging the photo-controlled charge trapping/detrapping process and the partially top-gated energy band landscape, the van der Waals heterostacking achieves polarity storage and logic reconfigurable characteristics, respectively. Precise polarity tunability, logic nonvolatility, robustness against high temperature (at 85°C), and near-ideal subthreshold swing (80 mV dec^−1^) can be done. A comprehensive investigation of dynamic charge fluctuations provides a holistic understanding of the origins of nonvolatile reconfigurability (a trap level of 10^13^ cm^−2^ eV^−1^). Furthermore, we cascade such nonvolatile reconfigurable units into a monolithic circuit layer to demonstrate logic-in-memory computing possibilities, such as high-gain (65 at *V*_dd_ = 0.5 V) logic gates. This work provides an innovative 3D heterointegration prototype for future computing-in-memory hardware.

## Teaser

A 2D/3D heterostructure showcases nonvolatile and polarity-controllable functions.

## INTRODUCTION

Conventional computing schemes based on von Neumann’s architecture are facing challenges due to the growing computational demand in modern artificial intelligence technology (*1*–*3*). In-memory computing architecture that empowers to overcome the physical gap between memory and process components is proposed as a promising solution to execute in situ machine learning (*4*–*6*). Various electronic devices with nonvolatile memory (NVM) features, such as resistive memristors, ferroelectric semiconductor transistors, phase change memories, or ionic liquid–gated transistors, have been explored to develop robust in-memory computing hardware with analog or digital types (*7*–*12*). A key consideration in device selection is effective area and energy design, coupled with silicon (Si) back-end-of-line (BEOL) compatibility, considering that emerging computing architectures now rely heavily on complementary metal-oxide semiconductor (CMOS) electronics for key functions. For instance, current analog in-memory computing systems often use Si-based digital computers/converters to complete the vector-matrix multiplication accelerator (*13*–*15*). On the other hand, digital in-memory computing, such as Boolean logic-in-memory computing, typically requires a high degree of device reconfigurability to simplify system design—a challenge that current NVM technologies have yet to adequately address.

A specific type of logic transformable device, named reconfigurable field effect transistor (RFET), distinguishes itself from its counterparts and stands out among its counterparts as the dual-gate structure renders the enhancement of runtime tunability of carrier concentration and conductive polarity transformation between p- and n-type (*16*–*18*). RFETs can be engineered through the elaborate design of the Schottky barrier and the selective engineering of carrier transport via electrostatic fields. The flexible polarity reconfigurability can potentially foster compact circuit design without compromising accurate logic functions. The original RFET concept was demonstrated in an axial Si nanowire heterostructure with independently gated Schottky contact regions (*19*, *20*). However, as the demand for high-density integration continues to rise, the ongoing reduction in device size appears to pose a challenge to its electrostatic gating capabilities (*21*–*23*). This, in turn, creates a challenge to the device’s reconfiguring efficiency.

The utilization of van der Waals (vdW) semiconductors in constructing RFET exhibits great potential to enhance electrostatic gating efficiency because of their atomic thickness and uniformity (*24*–*26*). Recent advances in vdW RFET research have made progress on integrating diverse functions within a single unit, including tunneling transistors, multiple diode regimes, and ternary logic (*27*–*32*). Recent studies have successfully incorporated nonvolatile conductance tunability into vdW RFETs, paving the way for a logic-in-memory prototype for digital computing architectures (*33*, *34*). Nonetheless, these nonvolatile RFETs (NRFETs) rely on a pluri-gate structure or a metal-ferroelectric-insulator-semiconductor structure to control channel conductance and charge storage. This scenario poses challenges to retention performance and Si compatibility due to the high thermal budget process. This further raises challenges in maintaining good device reliability and unlocking the full potential of vdW RFETs as the device performance is extremely sensitive to its gate-terminal controllability. Consequently, efficient computing approaches call for device configurations and mechanisms to enable efficient collocation of logic and memory functions.

In this article, we demonstrate a photo-assisted vdW NRFET design for logic-in-memory computing architecture by integrating an ambipolar vdW transistor and a two-dimensional (2D)/3D interface. The vdW heterostacking uses the interface between hexagonal boron nitride (h-BN) and SiO_2_ as a reversible reservoir to constantly accommodate photo-induced carriers. Further, the partially top-gated configuration introduces a stair-shaped energy band into the channel and governs the fast flow of a specific type of carrier. This results in desirable characteristics for logic-in-memory applications, such as photo-induced nonvolatility, robustness against harsh environments (at 85°C), precisely controllable polarity reconfigurability, and steep slope [subthreshold swing (SS) of 80 mV dec^−1^]. An in-depth investigation into the dynamic charge fluctuating process near the surface region is further delineated via holistic low-frequency noise analysis and local Kelvin probe force microscope imaging, confirming the universality of the 2D/3D component with Si BEOL compatibility. In consequence, we demonstrate a fully complementary inverter with a high gain of 65 at a low *V*_dd_ = 0.5 V and several logic gate circuits featuring logic in memory. These achievements mark a notable step toward efficient monolithic integration prototype for shaping the landscape of RFET-based logic-in-memory technologies.

## RESULTS

### Device of vdW NRFETs

**Fig. 1.**
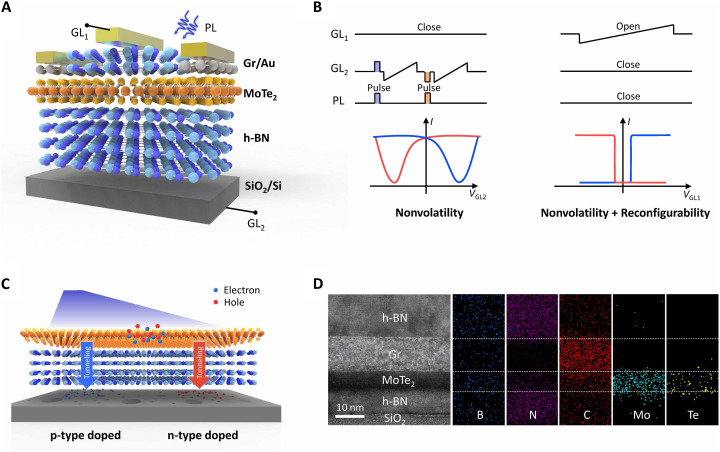
Nonvolatile vdW RFET. (**A**) Schematic illustration of nonvolatile vdW RFET, which consists of an ambipolar channel, a 2D/3D heterointegrated interface, and a partially covered top gate configuration. (**B**) Operating regime of a vdW NRFET with both nonvolatility and reconfigurability. GL_1_, GL_2_, and PL represent the top gate line, back gate line, and photo line. (**C**) Schematic of photo-induced polarity change (n-type doped/p-type doped) and nonvolatile mechanisms. (**D**) A cross-sectional STEM–High-Angle Annular Dark-Field (HAADF) image of an h-BN/Gr/MoTe_2_/h-BN/SiO_2_ heterostructure (source/drain electrode region) stacked on a Si substrate. The corresponding energy-dispersive x-ray spectroscopy elemental mappings consider the existing elements of B, N, C, Mo, and Te.

The vdW NRFET is fabricated leveraging the flexible stackability and defect engineering capabilities of the vdW family. Figure 1A depicts the schematic of the vdW stack, which comprises a top gate, an ambipolar transition metal chalcogenide semiconductor channel, a 2D/3D interface, and a bottom gate. Under the synergetic effect of the bottom electrostatic field and light illumination, the original transfer characteristics featured with ambipolarity are expected to be tuned between n- and p-type dominant states, as outlined in the operating sequence shown in Fig. 1B (i). The inherent or interfacial defects of the 2D/3D blockings serve as a natural reservoir for storing photo-generated electrons and holes (Fig. 1C). As a result, the light-induced polarity change can be precisely accurately resolved into multiple states and maintained and sustained over a long duration without compromising the gate tunability. The top gate regime, demonstrated in Fig. 1B (ii), further molds the device’s logic reconfigurability as the engineered energy band landscapes enable selective carrier injection. With these features, the designed vdW NRFET structure consolidates desirable merits for a logic-in-memory computing architecture—including light-induced nonvolatility, precise controllability, and in situ logic reconfigurability—all of which will be sequentially introduced.

Taking MoTe_2_-based NRFET as an example, it consists of a MoTe_2_/h-BN/SiO_2_ trilayer, a titanium/gold (Ti/Au), and an h-BN layer functioning as the top gate electrode and dielectric layer, a Si substrate as the back-gate electrode, and two graphene flakes (Gr) as the source/drain electrodes. Scanning transport electron microscopy (STEM) images and elemental mappings of the cross-sectional view for both the source/drain electrode region (Fig. 1D) and the channel region (fig. S1) display the well-distinguished multilayer, indicating the good crystal uniformity of the fabricated device (fig. S1). The corresponding film thicknesses of Gr, MoTe_2_, bottom h-BN, and top h-BN are 18, 3, 10, and 17 nm, respectively (see fig. S2). The Raman spectrum collected from the device highlights typical Raman vibrational peaks: *E*_2g_ at 1366 cm^−1^ for h-BN, *B*^1^_2g_ at 171 cm^−1^, *A*^1^_g_ at ~232 cm^−1^, *E*^1^_2g_ at ~288 cm^−1^ for MoTe_2_, G at ~1581 cm^−1^, and 2D at ~2917 cm^−1^ for Gr (fig. S3) (*35*).

### Nonvolatile characteristics of the light-induced NRFET

**Fig. 2.**
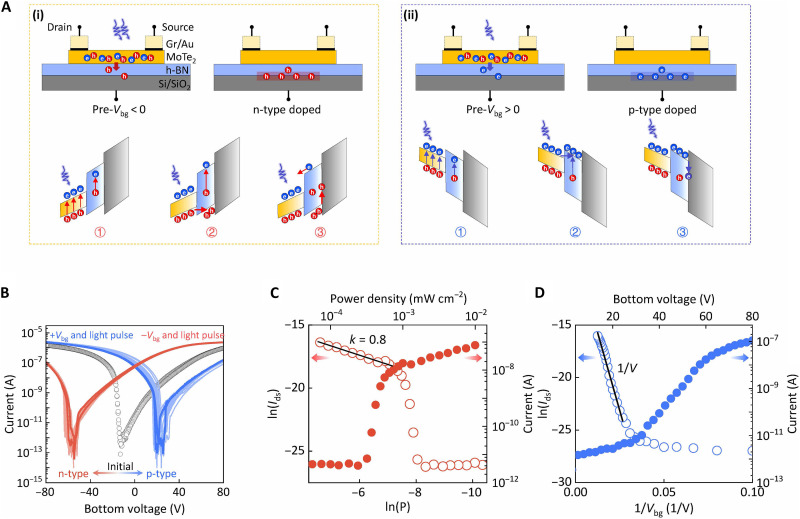
The photo-induced polarity change in MoTe_2_ NRFETs. (**A**) Schematic illustrations of the photo-induced doping behaviors and the corresponding energy band diagrams for the bottom-gated device at (i) n-type doped and (ii) p-type doped states. (**B**) Transfer characteristics of the bottom-gated device at its initial, n-type doped, and p-type doped states. (**C**) Readout current as a function of light power density after paired pulse of pre-*V*_bg_ and light illumination. A linear region can be observed when the power density is larger than 1 μW cm^−2^. (**D**) The readout current depends on the incident stimulus of paired pre-*V*_bg_ and light illumination. The fitted curve indicates a linear relationship between ln(*I*_ds_) and 1/*V*_bg_.

The nonvolatile light-induced polarity change in MoTe_2_ NRFET is first demonstrated under the bottom-gating regime, as the structure schematic shown in Fig. 2A. Initially, it exhibits typical ambipolar conduction behavior with the bottom-gate voltage (*V*_bg_) sweeping from −80 to 80 V (Fig. 2B). When the device is exposed to light illumination alongside the application of a preprocessing electric pulse (pre-*V*_bg_), a notable change in its conduction polarity is observed. As highlighted in Fig. 2B, a negative pre-*V*_bg_ leads to n-type dominant conduction and a positive one leads to p-type conduction, while no evident changes can be observed under a dark environment. This suggests that such a polarity change closely relates to the light signal. Specifically, the p-type and n-type transformation can be well repeated through a periodic set and reset operations via a pair of electric and light pulses, indicating the good reversibility of the photo-induced polarity change phenomenon. The time domain of the polarity control among the initial, n-type, and p-type conduction states visually confirms the reversible nonvolatility of the light-induced NRFET (fig. S4). Further quantification of the light tunability was undertaken by step-by-step tracking of the changing process of the device polarity. Figure 2C shows the incident light power density (*P*)–dependent readout current (*I*_ds_) at *V*_bg_ = 0 V. At *P* lower than 1 μW cm^−2^, the photo-generated carrier density is limited, leading to a slow change of the readout current. It then presents a steep linear relationship (*P* > 1 μW cm^−2^) between ln*I*_ds_ and ln*P* with a fitted slope (*k*) of around 0.8 after a pulse pair of light and *V*_bg_. Such a slope can be attributed to frequent charge trapping/detrapping events in this device (*36*–*38*). In addition, Fig. 2D plots the *I*_ds_ as a function of pre-*V*_bg_. When pre-*V*_bg_ exceeds 30 V, the readout current at *V*_bg_ = 0 linearly adheres to the variation of 1/*V*_bg_ (*39*–*42*). This scenario implies that a minimum of 30 V is required to create a narrow barrier for carriers to tunnel through. Consequently, we propose a benchmark for light-doping studies, considering the light/electrical signal–dependent readout current of the NRFET by *I*_ds_ ∝ *P^k^*exp[(*V*_bg_)^−1^].

An initial investigation into the origins of the photo-induced polarity change involves analyzing several specific structures. The transfer curves recorded for the bottom-gate device—excluding the bottom h-BN layer—almost remained unchanged following the paired stimulation of pre-*V*_bg_ and light illumination (fig. S5). This unveils that the main contributions are not from the MoTe_2_ channel or its top or bottom surface, instead confirming the synergetic role of the h-BN and SiO_2_ 2D/3D interface. Note that the difference between two initial states for device with and without bottom h-BN flake originates from the substrate effect, which is an invariance over the whole process. Further insights into the nonvolatile polarity change in MoTe_2_ NRFET can be gleaned from considering the charge transport process and energy band evolution grounded in interfacial trapping events. As shown in Fig. 2A, the charge flow driven by paired pulses can be described in three steps: (i) Under light illuminant, numerous electrons and holes are generated in the ambipolar channel; (ii) at the same time, the positive (negative) pre-*V*_bg_ drives the excited electrons (holes) to move into the h-BN/SiO_2_ interface via tunneling-dominant transport; (iii) the immersed electrons (holes) are trapped by inherent interfacial defects, leading to a permanent negative electrostatic field (positive electrostatic field), i.e., a p-type doping (n-type doping) effect on the conducting channel. This process rationalizes the nonvolatility of the light-induced polarity change in bottom-gated MoTe_2_ NRFET.

It is acknowledged that intrinsic defects with donor-like features are commonly found in h-BN flakes, and they play a supportive role in the polarity shift process (*43*–*46*). This can be understood by the sketches in Fig. 2A. Electrons in the defects of h-BN can be excited under light illumination and contribute to the photocurrent. The remaining charged ions would reinforce the positive electrostatic field under a negative pre-*V*_bg_ while partially counteracting the negative electrostatic field under a positive pre-*V*_bg_. Consequently, we can anticipate a stronger n-type doping behavior in h-BN–based NFET devices compared to the p-type branch. To visually confirm the doping effect of h-BN on MoTe_2_ NRFET device, a comparison device that consists of a thicker bottom h-BN layer (>20 nm) is investigated (fig. S6). As the thicker h-BN would largely inhibit contributions from 2D/3D interface, only an n-type doping behavior is observed. This phenomenon serves a dual purpose: It indirectly verifies the existence of donor-like defects in h-BN layers and elucidates the dominant role played by 2D/3D interfacial defects in achieving the reversible polarity change characteristics.

### The underlying mechanism of the nonvolatile characteristics

**Fig. 3.**
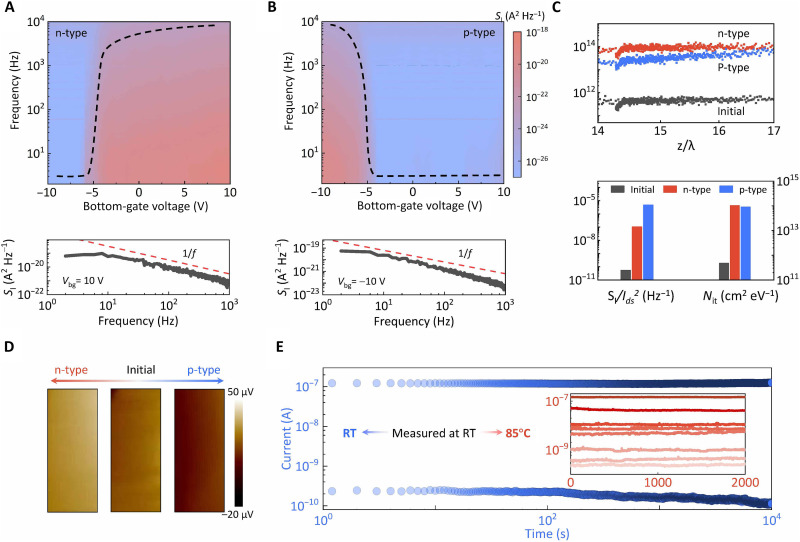
The origins of the photo-induced nonvolatile characteristic of MoTe_2_ NRFETs. (**A** and **B**) Mappings of current power spectrum density (*S_I_*) versus frequency and *V*_bg_ of MoTe_2_ NRFET at the n-type doped and p-type doped states, respectively. The dashed lines highlight the evolution of *S_I_* at a fixed frequency (*f* = 20 Hz). The bottom panel shows typical *S_I_* curves at *V*_bg_ = 10 V at these two states that follow the ideal 1/*f* signal (dashed line). (**C**) Top: Extracted *N*_it_ depending on *z*/λ for the MoTe_2_ NRFET under the three conditions, where *z* and λ represent the trap depth and the tunneling distance parameter, respectively. Bottom: Comparison plot of *S_I_*/*I*_ds_^2^ and *N*_it_ at *f* = 20 Hz at three states. (**D**) Evolution of the surface potential of MoTe_2_ NRFET via in situ KPFM characterization. (**E**) Retention performance of the device measured at room temperature (RT) and its multilevel readout currents measured at 85°C.

A clear physical mechanism is crucial for guiding performance optimization and commercial customization of electronic devices. To gain a visual understanding of how interfacial defects govern the nonvolatile polarity change in the NRFET, dynamic measurements were carried out using both holistic and local views via charge fluctuating noise and Kelvin Probe Force Microscopy (KPFM) technologies. Figure 3 (A and B) and fig. S7 show the mapping plot of the holistic current noise power spectral density (*S_I_*) of the device as a function of frequency and *V*_bg_ under different states. At a fixed voltage, the extracted *S_I_* curve versus frequency shows an ideal 1/*f* variation signal (Fig. 3, A and B, bottom). The initial log*S_I_* mapping and its transfer characteristic, as shown in fig. S7, exhibit a typical ambipolar behavior of the device. It changes to an electron (hole)–dominant conducting feature after the paired light and negative (positive) pre-*V*_bg_ stimuli, corresponding to the light-induced n-type (p-type) doped state. The evolution of *S_I_* profiles in Fig. 3 (A and B) mirrors the light-doped transfer characteristics and confirms the nonvolatile polarity change in MoTe_2_ NRFET. The frequency dependency can be defined according to the empirical formula *S_I_* ∝ $\frac{I_{\mathrm{ds}}^{\alpha }}{f^{\beta }}$, where α and β are the scaling exponents with the current and frequency, respectively (*47*, *48*). As provided in fig. S8, the fitted β for all three states near 1 indicates the existence of a uniform distribution of charge traps in space and energy, which can be attributed to the fact that the design of 2D/3D heterointegrated structure provides a flat interface for charge trapping/detrapping events. The extracted α values of 2 and *V*_ds_-independent *S_I_/I*_ds_^2^ manifest that the nonvolatile polarity change in the MoTe_2_ NRFET is rooted in the channel-surrounded trapping/detrapping phenomena instead of source/drain electrodes (fig. S9).

The physical images of the 1/*f* characteristics in MoTe_2_ NRFET are distinguished to profoundly discuss the charge fluctuating dynamics under different conditions. First, we found that the normalized $S_{I}/I_{\mathrm{ds}}^{2}$ as a function of (*g*_m_/*I*_ds_)^2^ follows the carrier number fluctuation model (*49*, *50*). This points out the governing role of the interfacial defects during the carrier transport process in a global view (fig. S10). The corresponding *N*_it_ that represents the total density of effective traps is further evaluated (Supplementary Note). As plotted in Fig. 3C, *N*_it_ shows a negligible fluctuation along with *z*/λ for three different conditions because of the uniform distribution of charge trapping/detrapping events in MoTe_2_ NRFET. The average values of *N*_it_ for n-doped and p-doped NRFET are 9.35 × 10^13^ and 3.82 × 10^13^ cm^−2^ eV^−1^, approximately two orders of magnitude larger than the initial state (5.16 × 10^11^ cm^−2^ eV^−1^). The difference can be understood that the paired light and electric stimuli enhance the charge trapping/releasing process between the 2D/3D interface and MoTe_2_ channel (Fig. 1C). This verifies our foregoing conjecture about the device mechanisms and confirms the heterointegrating design. Note that the slight difference between the n-doping level and p-doping level originates from the symmetric doping level given the intrinsic defects in h-BN layers, as shown in Fig. 2A.

In comparison to the low-frequency noise characterization that globally diagnoses the carrier fluctuations, KPFM characterization allows for the local examination of the nonvolatility of the light-doping phenomenon in MoTe_2_ NRFET. It is conducted by monitoring the in situ surface potential of the semiconductor channel (Fig. 3D). Following a paired pulse of pre-*V*_bg_ and light illumination, the recorded surface potential for a p-type (n-type) doped state is lower (higher) than its initial state. This slight reduction (increase) in surface potential is attributed to the charge trapping (detrapping) events from the MoTe_2_ channel (interfacial state) to the interfacial state (MoTe_2_ channel), as explained earlier. The retention performance of the device is further tested under harsh conditions to evaluate its robustness. At room temperature, the readout currents recorded under the n-type and p-type doped states at *V*_bg_ = 0 show a negligible fluctuation over 10^4^ s (Fig. 3A). This impressive retention performance can be replicated in eight different states at 85°C, underscoring its crucial suitability for memory devices operating in the typical environment (inset of Fig. 3A).

### Logic reconfigurable characteristic of the NRFET

**Fig. 4.**
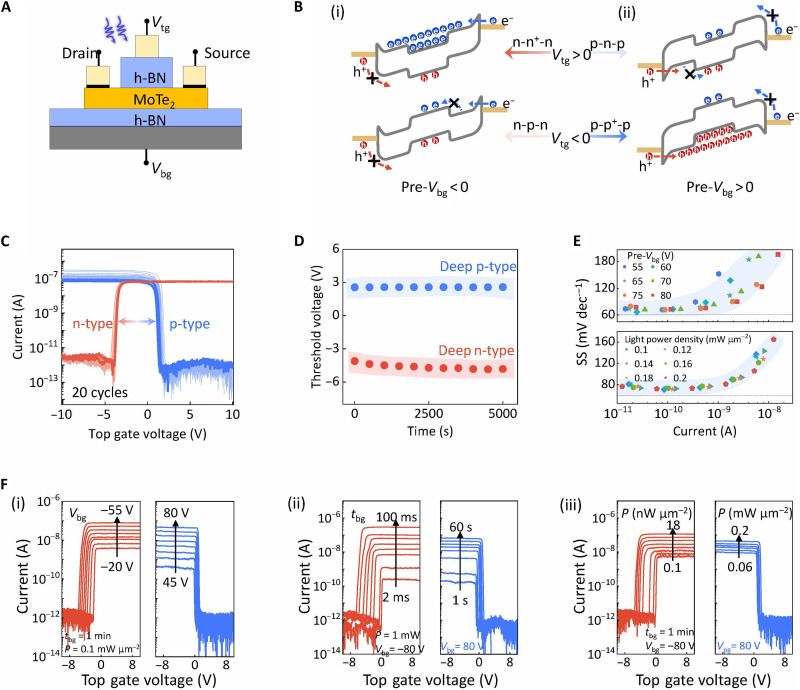
The reconfigurable characteristics of MoTe_2_ NRFETs. (**A**) Schematic of the dual-gated MoTe_2_ NRFET with a 2D/3D interface and a partially covered top gate configuration. (**B**) Corresponding energy band diagrams for 2D MoTe_2_ FETs under the modulation of partially covered *V*_tg_ at (i) n-type and (ii) p-type doped states. (**C**) Statistical transfer characteristics of the top-gated MoTe_2_ RFETs between n-type and p-type doped states. Each *I-V* curve are collected after preprogramming by *V*_bg_ and light stimulus. Sweeping the top gate terminal. (**D**) Retention test of the threshold voltage over 5000 s for the unipolar transfer characteristics under different conditions. (**E**) Extracted SS (taking n-type branch as an example) as a function of readout current depending on different pre-*V*_bg_ (top) and light power density (bottom). The dashed lines behave the value of 60 mV dec^−1^. (**F**) Multiple-level modulation of the reconfigurability of the device enabled by (i) pre-*V*_bg_, varying from −80 to −20 V for n-type and 45 to 80 V for p-type measurement; (ii) the pulse width of pre-*V*_bg_ (*t*_bg_), varying from 2 to 100 m for n-type and 1 to 60 s for p-type measurement; and (iii) incident light power density, varying from 0.1 to 18 nW μm^−2^ for n-type and 0.06 to 0.2 mW μm^−2^ for p-type measurement.

It is recognized that ambipolar conduction is undesirable in logic circuit applications due to the leakage current and the associated energy costs it incurs. In this regard, we exploit the electrostatic coupling of the top gate and MoTe_2_ channel to reshape the energy band landscape and refine the device configuration (Fig. 4A). With gate voltage (*V*_tg_) sweeping from −10 to 10 V, the recorded transfer characteristics after a pair of *V*_bg_ and light pulse exhibit typical n-type or p-type unipolar manner (Fig. 4C). A good symmetry and a high on-off current ratio above 10^6^ can be observed. The extracted threshold voltages as a function of time in Fig. 4F indicate that the conducting polarity is highly stable after releasing all external bias (Fig. 4D). The validity of the NRFET device concept is further confirmed by examining more than five devices (fig. S11). Such a scenario can also be reproduced in a purely electrical-operated MoTe_2_ RFET; however, this requires a constant gate bias (fig. S12). These results collectively demonstrate that both the nonvolatile and reconfigurable characteristics can be achieved in MoTe_2_ NRFET through the collaborative effect of the 2D/3D interfacial states, nonvolatile light doping, and dual-gate regime, making it more competitive in in situ logic computing architecture based on Si BEOL process (*7*, *51*, *52*).

The SS of a device is an index of its operating speed and applicable bandwidth, which are critical factors for logic circuits and computing technologies (*53*). The SS of the dual-gate MoTe_2_ NRFET is evaluated in Fig. 4E. For both the p-type and n-type branches, the work current rapidly switches when driven by *V*_tg_, leading to a good SS (80 mV dec^−1^, taking n-type branch as an example) near the thermionic limit. This indicates that no evident charge disturbance takes place near the semiconductor channel after the fulfillment of the light-induced charge trapping/releasing at the 2D/3D interface. The energy band evolution is resolved to understand the unipolar carrier transport in dual-gate MoTe_2_ NRFET (Fig. 4B). Distinct from the single globe bottom-gating configuration, a top gate placed in the middle divides the energy band of the MoTe_2_ channel into three zones. This configuration controls the electrical characteristic through both the Schottky barrier and the thermionic potential barrier (*26*, *54*). Taking panel (i) as an example, the channel is initially set to the n-type doped state under the paired pulse of negative pre-*V*_bg_ and light illumination. When applying a positive *V*_tg_, the middle zone of the MoTe_2_ channel bends downward, reshaping the energy band landscape of the entire channel into an n-n^+^-n configuration. The existing Schottky barrier and thermionic potential barrier hinder hole transport, resulting in high current in the n-type branch. In contrast, applying negative *V*_tg_ locally raises the energy band. This hinders the flow of electrons and holes due to the raised thermionic potential barrier in the middle zone and the Schottky barrier. This scenario leads to a completely off state compared to a typical ambipolar transistor. In consequence, a pure n-type conducting characteristic with a steep slope can be expected under the dual-gate configuration. Similarly, for a positive pre-*V*_bg_, holes dominate the MoTe_2_ channel. The positive (negative) *V*_tg_ enables a rare (intensive) carrier flow in the downward (upward) energy band, behaving as the pure p-type conduction. In this configuration, one can conclude that the 2D/3D heterointegration and the locally engineered top gate landscape endow the ambipolar vdW channel with nonvolatile and reconfigurable characteristics for in situ executing logic functions.

The tunable light-induced polarity change underpins the controllable reconfigurability of the device. As shown in Fig. 4F, incident signals ranging from the amplitude of pre-*V*_bg_, light pulse width, or light power density can modulate the readout current. This controllability originates from the fact that the amount of the trapped charges dominates the electrostatic light-doping level. These well-distinguished resistance states offer a broad engineering space to customize the threshold voltage of the devices for achieving desirable logic circuits. We would like to underscore that such an NRFET architecture, with nonvolatile and reconfigurable logic characteristics, has potential applications in other vdW stackings, such as WSe_2_-based NRFETs and ReSe_2_-based NRFET (fig. S13). These devices share common features such as a 2D/3D dielectric interface and an ambipolar channel, further confirming the robustness of the trap-related device mechanism and the universality of the device design.

### Demonstration of in situ logic computing

**Fig. 5.**
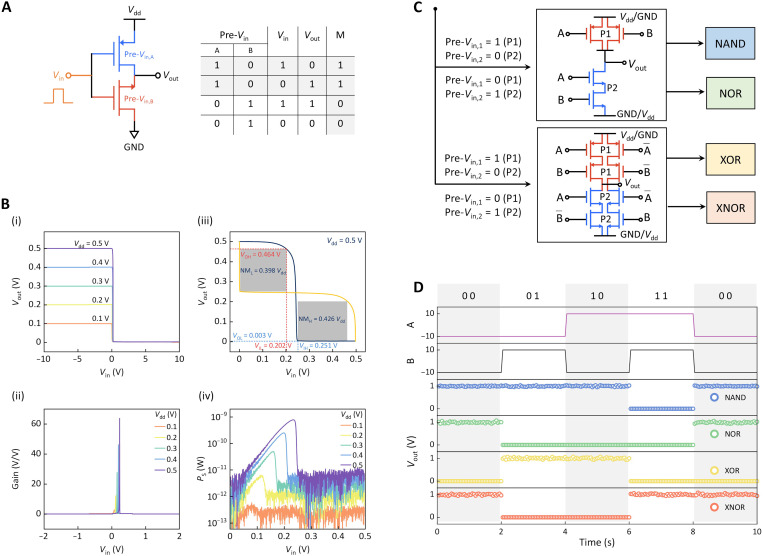
The demonstration of the logic-in-memory circuits based on MoTe_2_ NRFET arrays. (**A**) Schematic of the programmable inverter realized by two MoTe_2_ NRFETs on a monolithic channel. They are preprogrammed at n-type and p-type. (**B**) (i) The measured VTC of the inverter at different *V*_dd_ varying from 0.1 to 0.5 V. (ii) Calculated gain values as a function of input voltage (*V*_in_) at different *V*_dd_. (iii) Noise margin characteristics of the inverter at *V*_dd_ = 0.5 V. (iv) Calculated power consumption of the inverter versus *V*_in_ at different *V*_dd_. (**C**) Schematics of the programmable logic circuits and the corresponding two preprogramming information for NAND, NOR, XOR, and XNOR gates. (**D**) Simulated waveforms for operating various logic gate circuits.

The nonvolatility and reconfigurability of the conducting polarity endow NRFETs with the capability to implement in situ logic computing on a monolithic channel, i.e., monolithic logic circuit. It could potentially take over the traditional Si-based CMOS counterparts for developing logic computing technologies by circumventing certain complicated manufacturing processes, such as the definition of active regions. Moreover, the precise control over multiple levels of threshold voltage and conductance state allows the symmetric design of the p- and n-type transport characteristics, which is critical for fully leashing the performance of logic circuits. Figure 5A displays the built complementary inverter and its working states by a couple of MoTe_2_ NRFETs fabricated on a monolithic vdW channel. To achieve better inverter performance, they are carefully programmed as one p-type and one n-type devices with symmetric electrical characteristics, especially in terms of switching behavior (fig. S14), using two preprogramming signals (Pre-*V*_in,A_ and Pre-*V*_in,B_). After the preprogramming signals, the conducting states can be well maintained by the circuit, allowing half-storage of the logic results. Thus, we can use the voltage transfer characteristics (VTCs) to diagnose the previous information, i.e., the memory states of the circuit (M). Such a scenario demonstrates the logic-in-memory computing event. For example, a pair of signals of pre-*V*_in,A_ = logic 1 and pre-*V*_in,A_ = logic 0 are needed to enable the inverter function. After the operation, the pair pre-logic information is stored, corresponding to M = 1. As results shown in Fig. 5B, the VTC at various supply voltages (*V*_dd_) reveal the full-swing output operation. Such a VTC feature, coupled with ultralow SS and good conducting symmetry, leads to a high gain value of 65 at a maximal *V*_dd_ (0.5 V), which behaves the good amplification capability of the input signal and switching speed (Fig. 5B, ii). The ideal noise margin (NM) behavior of 0.43*V*_dd_ for NM_L_ and 0.43*V*_dd_ for NM_H_ discussed by mirroring the VTC reveals the robustness of the inverter against circuit noise (Fig. 5B, iii). Besides, Fig. 5B (iv) calculates the consumed power (*P*s) of the nonvolatile logic device–based inverter circuit using the formula of *P*s = *V*_dd_ × *I*_d_. A peak value of 770 pW at *V*_dd_ = 0.5 V reveals the importance of nonvolatility for implementing logic computing.

Typical logic gate circuits are further implemented by cascading NRFET arrays into a logic circuit layout. Figure 5C and fig. S15 display the circuit design of AND, OR, NAND, NOR, XOR, XNOR, and corresponding input preprogramming signals. For example, a typical NAND circuit is built by assembling two p-type NRFETs in parallel with two n-type NRFETs in series. Its simulated waveform is always logic 1 except when four input signals are all in high voltage (Fig. 5D). Such a circuit layout can also be used as a NOR gate by simply reversing the *V*_dd_ and Ground (GND) signals and reconfiguring the polarization of four NRFETs via electric and light pulses. Therefore, NRFET-enabled logic circuits allow multiple logic functions in one logic circuit, and the in situ storage of the input history substantially enhances the flexibility and efficiency of the circuit design in comparison to conventional CMOS technology.

The virtue of fast-speed switching in vdW NRFETs further encourages us to investigate the generalized in-memory process capability of NRFET-based analog circuits. Taking an output polarity controllable amplifier as an example, it was constructed by connecting a load resistor (*R*_load_ = 10 megohms) with a vdW RFET in series (fig. S16A). Input A (*V*_in,A_), which is the combination of light and *V*_bg_ signal, determines the conduction polarization of NRFET, and input B is a dynamic sinusoidal signal (*V*_in,B_) to apply to the top gate. For a negatively programmed input A (n-type), *V*_out,n_ showed the same phase as *V*_in,B_ (*V*_dd_ = 0.1 V), representing excellent common-drain output mode (blue line). For a positively programmed input A (p-type), a 180° phase difference can be observed between *V*_out,p_ and *V*_in,B_, corresponding to the common-source output mode (fig. S16B). In this scenario, input A can be in situ stored in the circuit, suggesting the signal processing capability of vdW NRFET-based analog circuits, such as phase-shift keying and frequency-shift keying.

## DISCUSSION

In summary, we have developed a logic-in-memory architecture by integrating nonvolatility and reconfigurability in one vdW stacking unit, which potentially offers a solution for the area- and energy-efficient computing challenges. The light-induced charge fluctuating behaviors between the ambipolar semiconductor channel and the 2D/3D interface are responsible for the nonvolatile characteristic of the NRFET devices, as visualized by the light-assisted dynamic charge trapping-detrapping process. The top-gated energy band landscape of the vdW channel further reshapes the logic reconfigurability of the device. Such scenarios concertedly enable excellent device performance for digital computing in reliable photo-induced logic nonvolatility and good controllability over dominant carrier type, threshold voltage, and multiple resistance states. These metrics can be universally observed in typical ambipolar channels, such as MoTe_2_, WSe_2_, and ReSe_2_, and endow the NRFET units with the capability for logic-in-memory computing on a 2D/3D monolithic heterointegrated structure. Consequently, this work showcases the remarkable adaptability and integration advantages of vdW heterostructures in shaping computing technologies.

## MATERIALS AND METHODS

### Device fabrication

The vdW NRFETs were prepared by mechanical exfoliation and dry-transfer method. Taking the heterostructure of h-BN/Gr/MoTe_2_/h-BN as an example, it was stacked layer by layer on a Si substrate with a 300-nm-thick SiO_2_ layer to form the 2D/3D heterointegration. Then, the top gate, source, and drain electrodes (Ti/Au: 15/50 nm thick) were defined by electron beam lithography and thermal evaporation.

### Characterization

The morphology and thickness were measured by optical microscopy (BX53M microscope with DP26 digital camera; Olympus Corp.) and atomic force microscopy (AFM; Solar TII; Tokyo Instruments Inc.). Raman spectroscopy was performed with an excitation wavelength of 532 nm (Nanofinder 30 with 523-nm excitation laser; Tokyo Instruments Inc.). The device morphology and its microstructure were further examined using a field-emission transmission electron microscope (JEM-F200; JEOL Corp.), equipped with an energy-dispersive x-ray spectroscopy system.

### Device properties

Electrical characterization of the MoTe_2_ NRFET was performed in a probe station (TTPX, Lake Shore Cryotronics Inc.) equipped with a semiconductor parameter analyzer (Keysight, B1500A). Optoelectrical properties of the devices were characterized under an ultralong-distance laser beam–shaping module (JadeDot-LDPS, Southport Corp.) combined with an oscilloscope (Keysight DSOX2024a) to modulate light illuminant. The wavelength of the selected laser is 445 nm. To prevent unnecessary fluctuations caused by the environment, all the electrical measurements were performed in a vacuum (<10^−5^ torr). The dynamic charge characteristic measurements were performed on the basis of a programmable point probe noise measurement system (3PNMS, Synergie Concept) with a system noise floor of 10^−27^ A^2^ Hz^−1^. The current fluctuations of the device were recorded at a certain bottom-gate voltage and a source-drain voltage under the initial state, n-type doped state (a paired pulse of light illumination and −*V*_bg_), and p-type doped state (a paired pulse of light illumination and +*V*_bg_), respectively. For in situ KPFM measurements, a Bruker Dimension Icon SPM system was connected with an external precision source unit (Keysight B2912A) to provide a bottom-gate bias pulse. The Pt/Ir conductive AFM probe was used to monitor the variation of the surface potential under the tapping mode.

## Supplementary Materials

### This PDF file includes:

Figs. S1 to S16 Supplementary Note
