# Supplementary material for: Silicon–van der Waals heterointegration for CMOS-compatible logic-in-memory design
Source: Sci Adv. 2023 Dec 8;9(49):eadk1597. doi: 10.1126/sciadv.adk1597 (PMC10708197; doi:10.1126/sciadv.adk1597)
Supplement: Supplementary file 1 — Figs. S1 to S16 Supplementary Note [file sciadv.adk1597_sm.pdf]

Science Advances
AAAS

# Supplementary Materials for

## Silicon–van der Waals heterointegration for CMOS-compatible logic-in-memory design

Mu-Pai Lee *et al.*

Corresponding author: Wenwu Li, liwenwu@fudan.edu.cn; Mengjiao Li, mjli@shu.edu.cn; Wen-Wei Wu, wwwu@nycu.edu.tw; Yen-Fu Lin, yenfulin@nchu.edu.tw

*Sci. Adv.* **9**, eadk1597 (2023)
DOI: 10.1126/sciadv.adk1597

**This PDF file includes:**

Figs. S1 to S16
Supplementary Note

# Supplementary Information

## Silicon-van der Waals heterointegration for CMOS-compatible logic-in-memory design

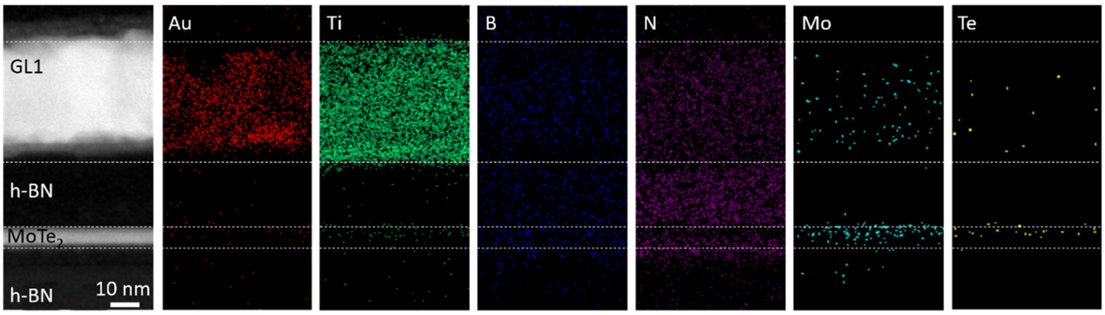


**Figure S1 STEM characterizations.** A cross-sectional STEM-HAADF image of the h-BN/$MoTe_2$/h-BN/$SiO_2$ heterostructure (channel region) stacking on a Si substrate. Scale bar: 10 nm. The corresponding EDS elemental mappings reveal the compositional elements of Au, Ti, B, N, Mo, and Te.

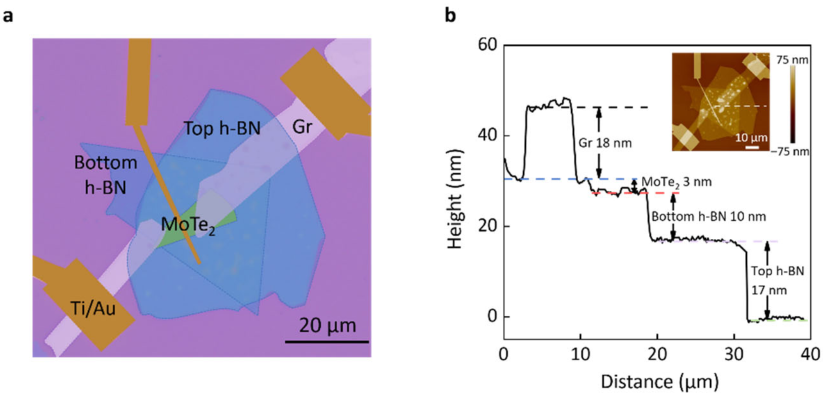


**Figure S2 Device architecture and thickness characterizations. a.** Optical microscope image of the vdW NRFET that consists of a top electrode, a $MoTe_2$ channel, a 2D/3D (h-BN/$SiO_2$) heterostructure, and a bottom gate. **b** AFM image revealing the typical thicknesses of Gr, $MoTe_2$, bottom h-BN, and top h-BN flakes.

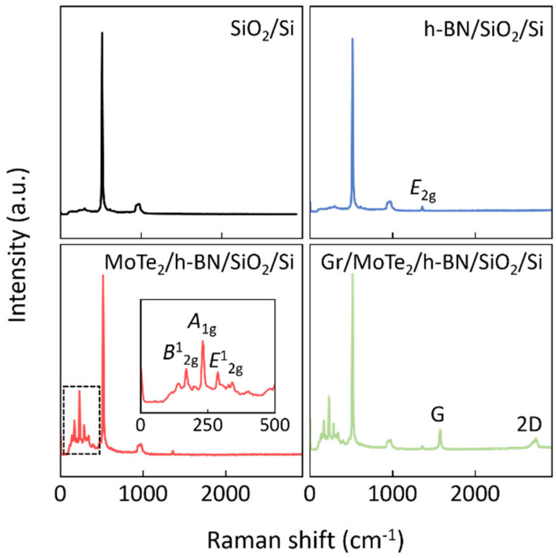


**Figure S3 Raman characterizations.** Raman spectra of the $SiO_2$/Si, h-BN, $MoTe_2$/h-BN, Gr/$MoTe_2$/h-BN heterostackings on $SiO_2$/Si substrate.

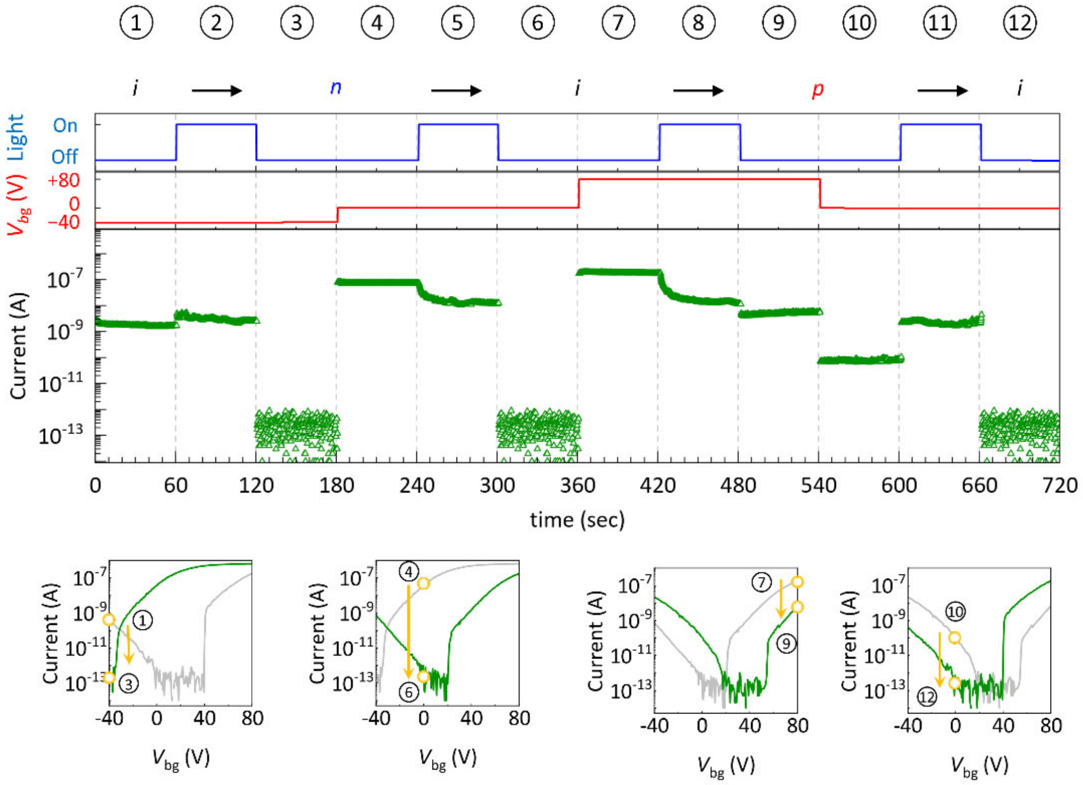


**Figure S4 Time domain of the charge polarity control.** a. Time domain of electrical behavior transformation among initial state, n-type doping state, and p-type doping state. b. The recorded transfer curves for various conditions, including the transition from the initial state to n-type doping, from n-type doping back to the initial state, from the initial state to p-type doping, and then from p-type doping back to the initial state.

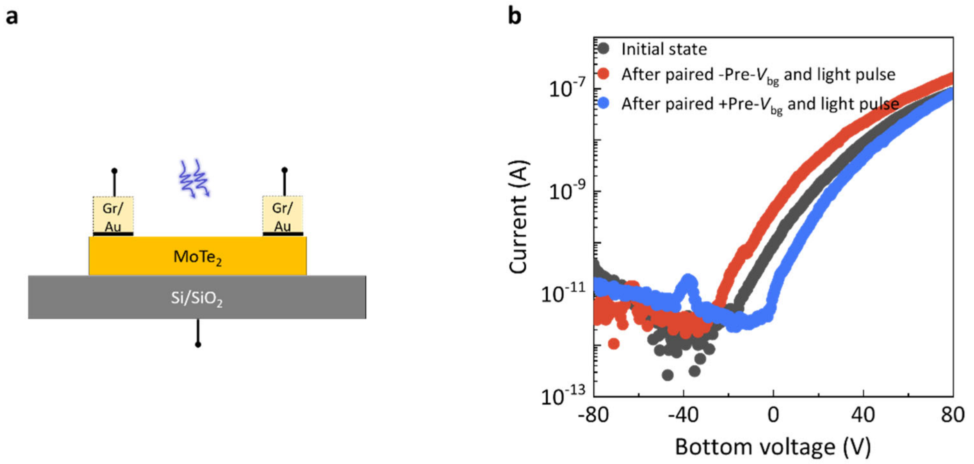


**Figure S5 Device structure and transfer characterizations of the $MoTe_2$ transistor** **a.** Schematic structure of the $MoTe_2$ transistor without h-BN ($MoTe_2$ w/o h-BN). **b.** The transfer curves of $MoTe_2$ w/o h-BN transistor as functions of $V_{bg}$ under its initial state, after paired negative pre-$V_{bg}$ and light pulse, and after paired positive pre-$V_{bg}$ and light pulse. The small difference among them indicates the important role of bottom h-BN in NRFET.

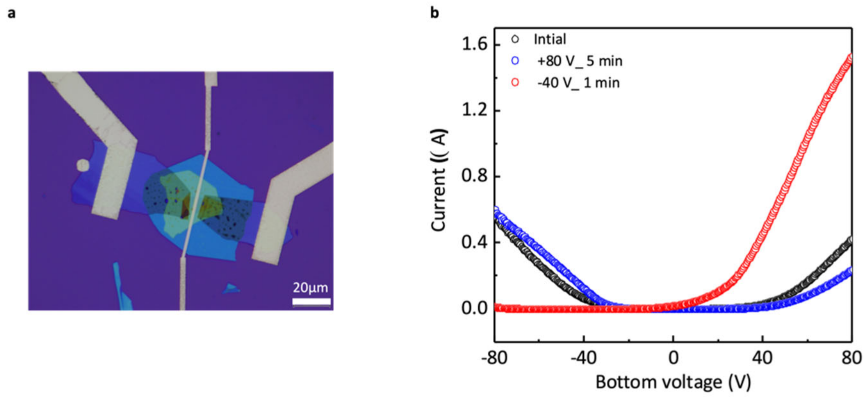


**Figure S6 Device architecture and transfer characterizations of a thick-BN NRFET. a.** Optical microscopy image of the fabricated Thick-BN NRFET device. **b.** The electrical characteristics of the Thick-BN NRFET device under different states. The corresponding conditions are marked and the applied $V_{ds}$ is 0.1 V.

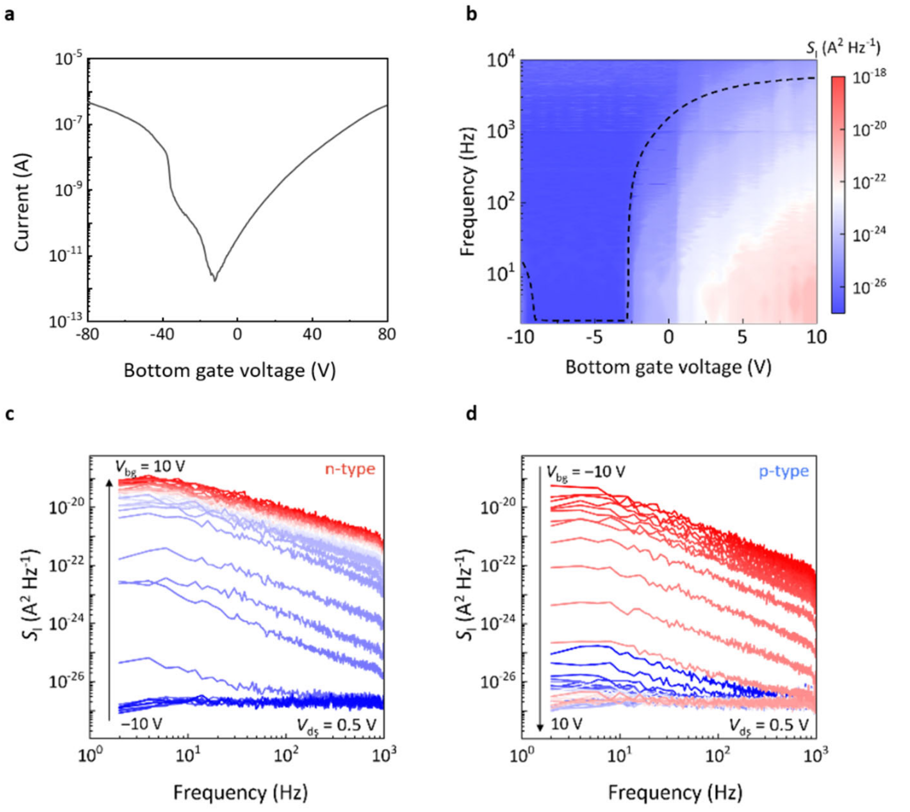


**Figure S7 Dynamic characterizations of the NRFET. a.** Transfer curve of a $MoTe_2$ NRFET under its initial state. Drain current power spectrum density depending on the frequency and $V_{bg}$ of the $MoTe_2$ NRFET for **b.** its initial (mapping plot), **c.** n-type, and **d.** p-type state at $V_{ds}$ = 0.5 V. The dashed line in panel **b** highlights the initial transfer curve of the device.

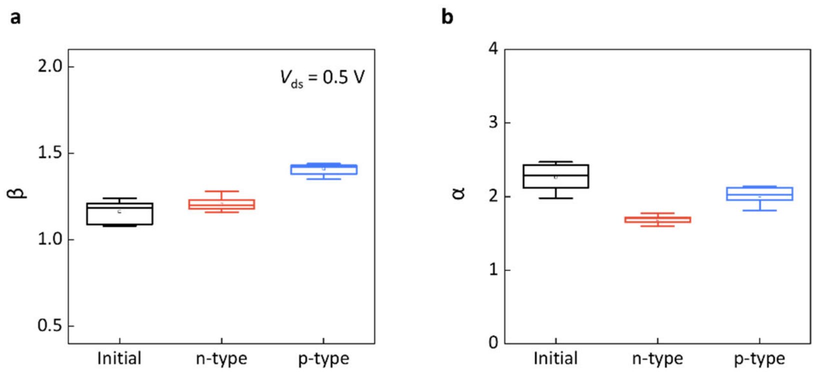


**Figure S8 Parameter analyses of charge trapping/detrapping events.** Box plots of the fitted **a** $\beta$ and **b** $\alpha$ values in $MoTe_2$ RFET for initial, n-type, and p-type states. Note that the center line, the upper, and the lower bars correspond to the average, the maximum, and the minimum values, respectively.

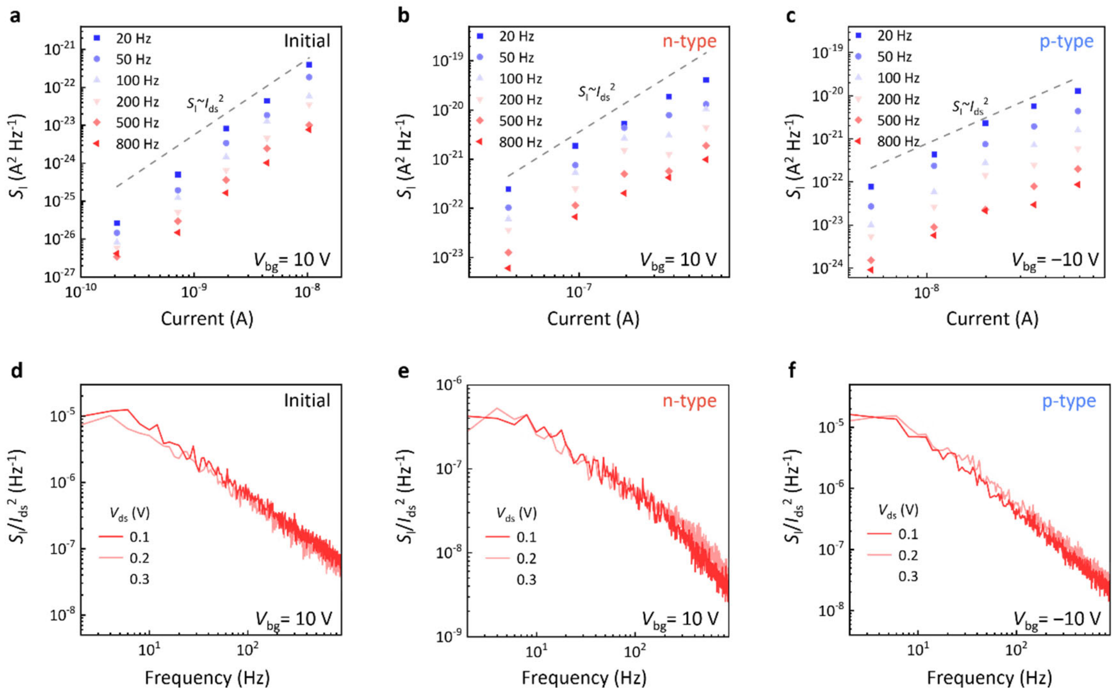


**Figure S9 Current fluctuations for different polarities.** Typical $S_I$ in $MoTe_2$ NRFET as a function of $I_{ds}$ at different frequencies for **a.** initial, **b.** n-type, and **c.** p-type state. The dashed line shows a typical $S_I \sim I_{ds}^2$ trend for reference. The normalized $S_I$ by $I_{ds}^2$ as a function of frequency at **d.** initial, **e.** n-type, and **f.** p-type states.

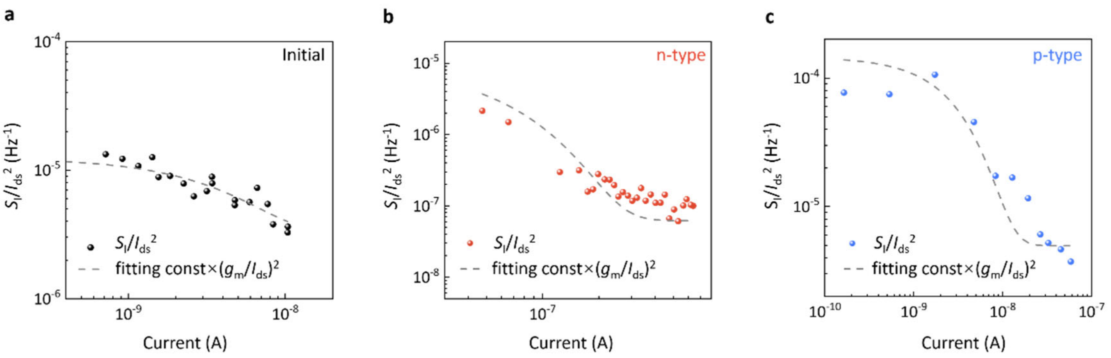


**Figure S10 Normalized power spectral densities of different polarties.** The comparison plot of $S_I/I_{ds}^2$ versus $I_{ds}$ at $f$ = 20 Hz and a constant×$(g_m/I_{ds})^2$ under different states. The value of the constant was adjusted to fit the data.

**Supplementary Note**

Typical carrier fluctuation models in nanoscale electronics include carrier number fluctuation (CNF) and Hooge mobility fluctuation (HMF). For these two modes, the dynamic carrier trapping/detrapping events can be assigned as the interfacial defects-dominant effect and bulk photon scattering effect, respectively. The charge fluctuation behavior in the proposed NRFET is diagnosed as CNF model by plotting the $S_I/I_{ds}^2$ versus Ids in log-log scale. The density of the effective traps ($N_{it}$) can be further extracted according to the following functions. For interfacial dominant current fluctuation, the normalized $S_I$ can be defined by the expression $\frac{S_I}{I_{ds}^2} = S_{Vfb}(\frac{g_m}{I_{ds}})^2$, where $S_{Vfb} = \frac{q^2 k_B T N_{it}}{fWLC_{ox}^2}$, is the flat-band voltage spectral density, and $q$, $k_B$, $T$, $C_{ox}$, $W$, $L$, and $g_m$ are the elementary charge, Boltzmann constant, absolute temperature in kelvin, gate capacitance per unit area (F cm$^{-2}$), channel width, channel length, and gate transconductance, respectively. Thus, as shown in Figure S10, $N_{it}$ can be obtained at different conditions by fitting the slope of $g_m/I_{ds}^2$ (dashed lines).

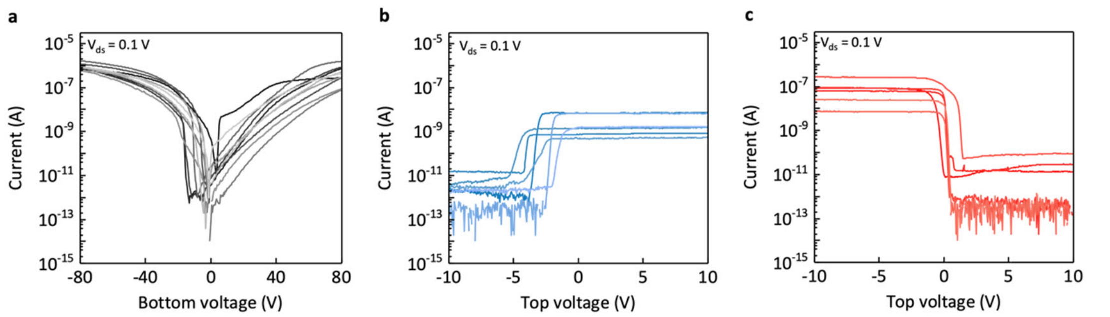


**Figure S11 Confirmation of study validity.** Electrical characteristics of six NRFET devices under various states.

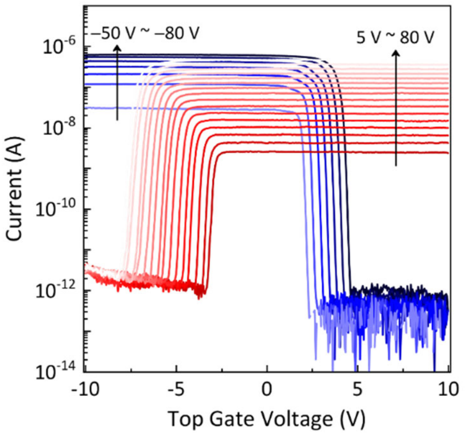


**Figure S12 Electrical-operated $MoTe_2$ RFET.** The transfer characteristics of $MoTe_2$ NRFET under the pure electric operation.

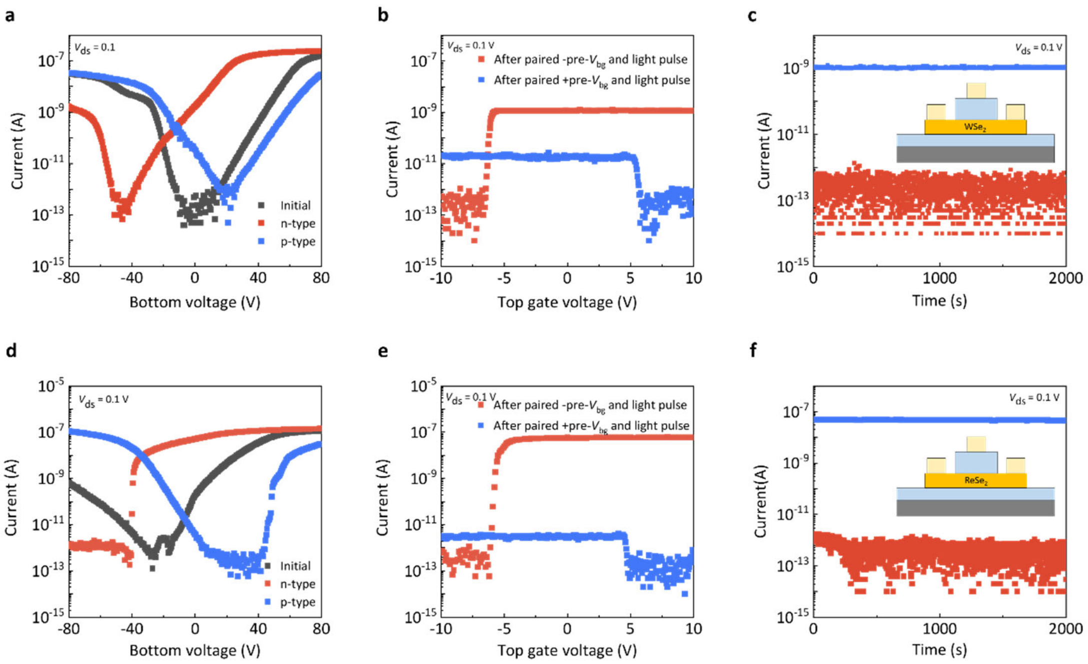


**Figure S13 Universality of the device design.** The electrical characteristics of $WSe_2$ and $ReSe_2$ NRFETs, including the bottom-gate transfer curve, the dual gate transfer curve, and the retention behavior for (a-c) $WSe_2$ and (d-f) $ReSe_2$ NRFETs, respectively.

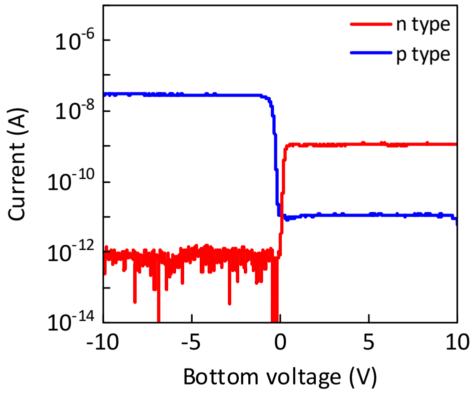


**Figure S14 Realization of inverter functions.** The transfer characteristics of two programmed $MoTe_2$ NRFET units for inverter application, featuring symmetric threshold voltage and asymmetric working current.

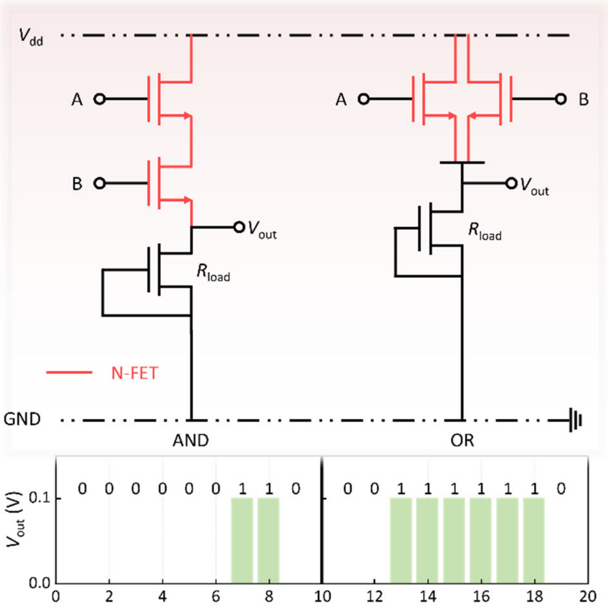


**Figure S15 NRFET-based logic circuits.** Schematic and corresponding $V_{out}$ waveforms of AND, and OR logic circuits.

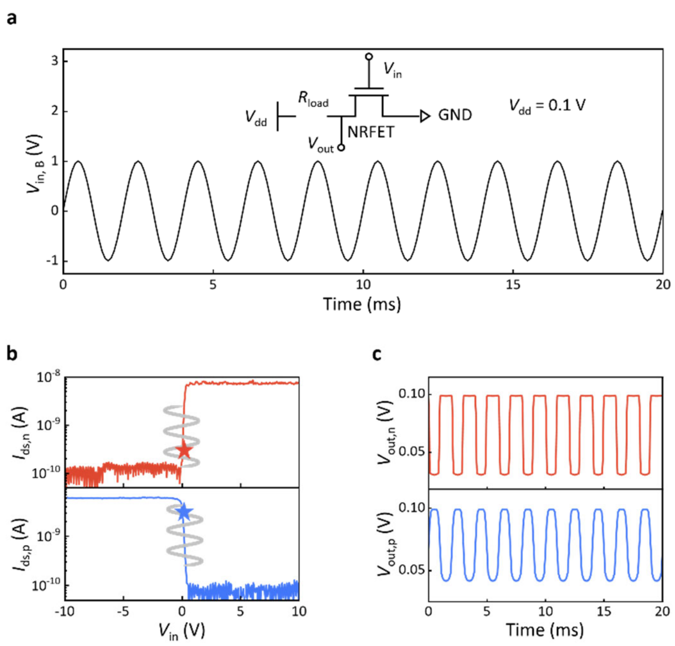


**Figure S16 NRFET-based analog circuits. a.** Time trace $V_{in,A}$ for the operation of the OPC amplifier. Inset shows the schematic of the OPC amplifier consisting of a load resistor ($R_{load}$) and an NRFET in series. **b.** Typical n-type and p-type transfer curves from the configurable $MoTe_2$ RFET with good symmetry. A weak sinusoidal signal $V_{ac}$ was superimposed at a fixed $V_{bg}$ (0 V). **c.** Time-tracked $V_{out,n}$ (upper) and $V_{out,p}$ (lower) for the operation of the OPC amplifier under common-drain (top panel) and common-source (bottom panel) modes.
